# Open-channel blockade is less effective on GluN3B than GluN3A subunit-containing NMDA receptors

**DOI:** 10.1016/j.ejphar.2012.04.036

**Published:** 2012-07-05

**Authors:** David W. McClymont, John Harris, Ian R. Mellor

**Affiliations:** a School of Biology, University of Nottingham, University Park, Nottingham, UK; b School of Biosciences, University of Nottingham, Sutton Bonington Campus, Loughborough, UK

**Keywords:** *N*-methyl-d-aspartate receptors, GluN3 subunit, Channel block, Magnesium, Memantine, MK-801, Philanthotoxin-343, Methoctramine

## Abstract

The GluN3 subunits of the *N*-methyl-d-aspartate (NMDA) receptor are known to reduce its Ca^2+^ permeability and Mg^2+^ sensitivity, however, little is known about their effects on other channel blockers. cRNAs for rat NMDA receptor subunits were injected into *Xenopus* oocytes and responses to NMDA and glycine were recorded using two electrode voltage clamp. Channel block of receptors containing GluN1-1a/2A, GluN1-1a/2A/3A or GluN1-1a/2A/3B subunits was characterised using Mg^2+^, memantine, MK-801, philanthotoxin-343 and methoctramine. IC_50_ values for Mg^2+^ and memantine increased when receptors contained GluN3A subunits and were further increased when they contained GluN3B, e.g. IC_50_s at − 75 mV for block of GluN1-1a/2A, GluN1-1a/2A/3A and GluN1-1a/2A/3B receptors respectively were 4.2, 22.4 and 40.1 μM for Mg^2+^, and 2.5, 7.5 and 17.5 μM for memantine. Blocking activity was found to be fully or partially restored when G or R (at the N and N + 1 sites respectively) were mutated to N in GluN3A. Thus, the changes cannot be attributed to the loss of the N or N + 1 sites alone, but rather involve both sites or residues elsewhere. Block by MK-801 and philanthotoxin-343 was also reduced by GluN3A, most strongly at − 100 mV but not at − 50 mV, and by GluN3B at all V_h_. Methoctramine was the least sensitive to introduction of GluN3 subunits suggesting a minimal interaction with the N and N + 1 sites. We conclude that GluN3B-containing receptors provide increased resistance to channel block compared to GluN3A-containing receptors and this must be due to differences outside the deep pore region (N site and deeper).

## Graphical abstract

**Figure:**
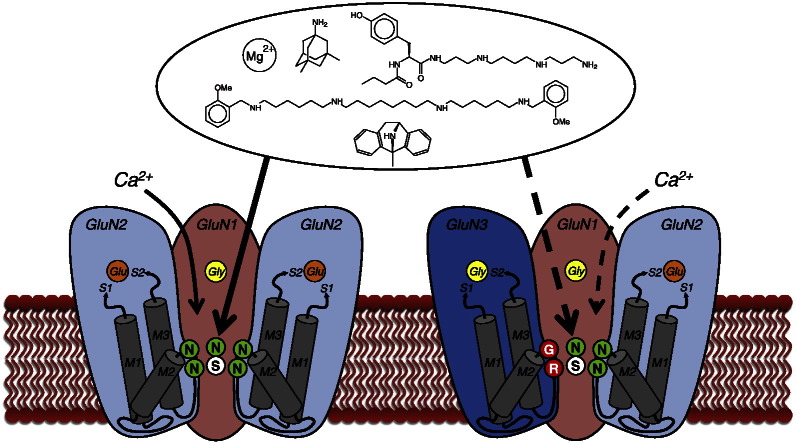

## 1 Introduction

*N*-methyl-d-aspartate (NMDA) receptors are a subgroup of the ionotropic glutamate receptor family (Traynelis et al., 2010) that are the products of seven genes giving rise to GluN1, GluN2A-D and GluN3A-B subunits. GluN1 undergoes variable splicing producing eight variants (GluN1-1a to GluN1-4a and GluN1-1b to 1-4b) (Dingledine et al., 1999). Most NMDA receptors are formed by the assembly of two GluN1 subunits and two GluN2 subunits in a 1/2/1/2 orientation forming a pore (Sobolevsky et al., 2009). GluN2 is activated by glutamate while GluN1 is activated by glycine. GluN3 may combine with GluN1 and GluN2 to form a functional receptor where, like GluN1, it contributes a glycine binding site.

**Fig. 1:**
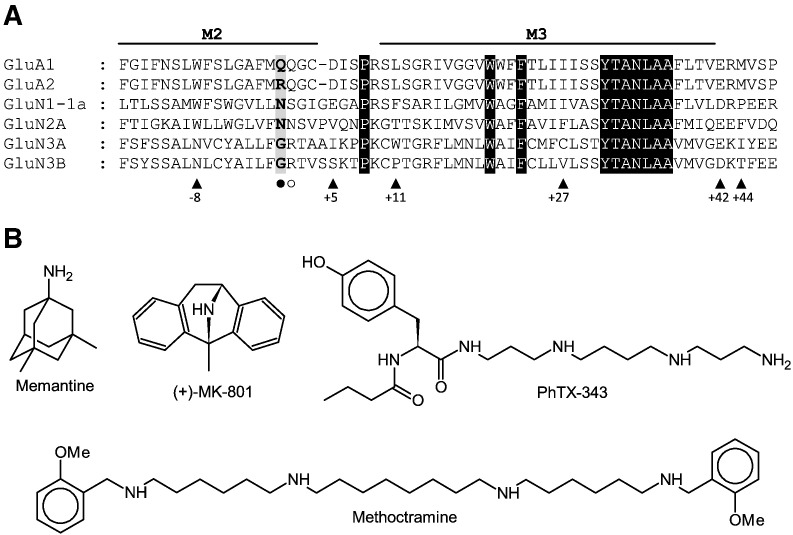
Comparison of ionotropic glutamate receptor sequences and NMDA receptor antagonists used in this study. A, Sequence alignment of the M2-M3 regions of GluA1, GluA2, GluN1-1a, GluN2A, GluN3A and GluN3B subunits. Highlighted with ● is the Q/R/N site and ○ is the N + 1 site. Other amino acids of interest are indicated by ▲ and numbered by their relationship to the N site. Highlighted in black are conserved amino acids. B, 2-D structures of memantine, (+)-MK-801, PhTX-343 and methoctramine.

The GluN3 subunits are thought to act in a dominant negative manner reducing block by Mg^2+^ and Ca^2+^ permeability (Sasaki et al., 2002). Differences may be due to amino acid changes at the so called ‘N site’ selectivity filter, known to be a binding site for Mg^2+^ (Wollmuth et al., 1998); this is N in both GluN1 and GluN2 subunits but G in GluN3 subunits (● in Fig. 1A). Additionally, the N + 1 site, also thought to play a role in Mg^2+^ block, becomes a positively charged R in GluN3 subunits rather than S or N in GluN1 and GluN2 respectively (○ in Fig. 1A).

There have also been reports of glycine gated receptors that are formed from GluN1 and GluN3 subunits following expression in *Xenopus* oocytes (Chatterton et al., 2002); these receptors have been found on white matter of the optic nerve (Pina-Crespo et al., 2010), but it is not yet clear if they exist in neurones.

The activity of open-channel blockers on GluN3 containing receptors has yet to be fully explored. The IC_50_ for Mg^2+^ has been shown to increase 14-fold in transgenic mice over-expressing GluN3A compared with controls (Tong et al., 2008). Other studies investigating Mg^2+^ block have only used a small range of concentrations with variable outcomes, possibly because they have employed different subunit combinations and expression systems (Cavara et al., 2010; Sasaki et al., 2002; Yamakura et al., 2005). Further investigation of the endogenous blocker is necessary to generate a more complete picture of Mg^2+^ block of different GluN3-containing receptors which is important for interpreting function *in vivo*. It is also currently unknown if the well-known NMDA receptor channel blockers memantine and MK-801 are influenced by GluN3 subunits or whether block by the polyamine-containing philanthotoxins (PhTXs) is reduced in a similar manner as with AMPA receptors, where an R in the pore prevents block (Brackley et al., 1993).

Here we aim to further characterise how GluN3 subunits influence block of NMDA receptors by Mg^2+^, memantine, (+)-MK-801 and PhTX-343 (Fig. 1B), while doing a direct comparison between GluN3A and GluN3B may help elucidate their role *in vivo*. We also include another polyamine-containing compound, methoctramine (Fig. 1B), a weakly voltage-dependent inhibitor of NMDA receptors in rat cortical neurones (that also blocks muscarinic and nicotinic acetylcholine receptors, and TRPV1 channels), that has not been tested at recombinant NMDA receptors (Melchiorre et al., 2003; Mellor et al., 2004).

## 2 Materials and methods

### 2.1 Materials

PhTX-343 was a gift from Professors Jerzy Jaroszewski and Kristian Strømgaard, Department of Medicinal Chemistry, University of Copenhagen, Denmark. Methoctramine was a gift from Professors Carlo Melchiorre and Anna Minarini, Department of Pharmaceutical Sciences, University of Bologna, Italy. All other antagonists (memantine, (+)-MK-801) and reagents were from Sigma-Aldrich-RBI (Poole, UK). NMDA was from Ascent Scientific (Bristol, UK).

### 2.2 Molecular biology

NMDA receptor clones used were GluN1-1a and GluN2A in pRK7, and GluN3A and GluN3B in pcDNA3.1 (gift from Dr. Dongxian Zhang, Burnham Institute, La Jolla, CA), which were confirmed by sequencing. Mutations were introduced using the QuikChange site-directed mutagenesis kit (Stratagene Cloning Systems, UK). Wild-type and mutant plasmids were linearised using the appropriate restriction enzyme and cRNA transcripts were synthesised using the mMESSAGE mMACHINE kit (Ambion). Transcripts were dissolved in H_2_O to a final concentration of 1 μg/μl and stored at − 80 °C until required.

### 2.3 Oocyte preparation

Oocytes were obtained by ovariectomy of female *Xenopus laevis* (European Xenopus Resource Centre, University of Portsmouth, UK) anesthetized with 2 g/L ethyl 3-aminobenzoate methanesulfonic acid (MS-222). The animals were humanely killed before oocyte collection in order to obtain schedule 1 status for the study. This protocol was approved by the Committee on Animal Resources, University of Nottingham. Oocytes were prepared for injection by treating fragments of ovary with 0.2 mg/ml collagenase (type 1A, Sigma) in Ca^2+^-free GTP solution (96 mM NaCl, 2 mM KCl, 1 mM MgCl_2_, 2.5 mM Na-pyruvate, 0.5 mM theophylline, 50 mg ml^− 1^ gentamicin sulfate and 5 mM HEPES, pH 7.5 with NaOH) for 1–2 h followed by thorough rinsing. cRNA transcripts of either WT or mutant subunits were mixed in a 1:1 ratio by weight for GluN1-1a/GluN2A or 1:1:3 ratio for GluN1-1a/GluN2A/GluN3(A or B) to give a minimum concentration of 50 ng/μL. Oocytes were injected with 50 nl of the mixture, and then incubated at 19 °C for 48 h in GTP solution (96 mM NaCl, 2 mM KCl, 1.8 mM CaCl_2_, 1 mM MgCl_2_, 2.5 mM Na-pyruvate, 0.5 mM theophylline, 50 mg ml^− 1^ gentamicin sulfate and 5 mM HEPES, pH 7.5 with NaOH) prior to experimentation.

### 2.4 Electrophysiology

Single oocytes were transferred to a perfusion bath and continuously washed with saline containing 95 mM NaCl, 2 mM KCl, 2 mM CaCl_2_, 5 mM HEPES, pH 7.5 with NaOH. Microelectrodes were pulled from borosilicate glass capillaries (TW150F-4, World Precision Instruments) using a Sutter P-97 programmable puller and had resistances of ~ 0.5 MΩ when filled with 3.0 M KCl. The oocytes were voltage clamped at holding potentials (V_h_) in the range of 0 to -100 mV using an Axoclamp 2A (Axon Instruments), and output currents were transferred to a PC using a CED 1401 plus interface (Cambridge Electronic Design) and WinEDR software (Dr. John Dempster, Institute of Pharmacy and Biomedical Sciences, University of Strathclyde, UK). To assess antagonist potencies, responses of NMDA receptors were elicited by perfusion of saturating concentrations of NMDA and glycine in the absence and presence of test compounds in the range of 0.01 to 100 μM. Once the response to NMDA/glycine had stabilized, antagonist concentrations were applied sequentially (lowest to highest) until a new stable current was obtained (in the absence of active NMDA receptor antagonists, the current remains unaffected during this application regime; Supplementary Fig. 1). Currents in the presence of antagonist were normalized to the control current in the absence of antagonist (= 100%). IC_50_ values were obtained by fitting antagonist concentration–inhibition curves by the equation:

$$\%\text{ control response}=100/\left(1+{\left({\text{IC}}_{50}/\left[\text{B}\right]\right)}^{\text{S}}\right)$$

where [B] is the antagonist concentration and S is the Hill slope.

Curves were fit using Graphpad Prism 5 and IC_50_ values were compared by an extra sum-of-squares F‐test with differences considered significant for *P* < 0.05. The influence of GluN3 on the current in response to NMDA/glycine was compared using one-way ANOVA followed by Bonferroni post-hoc comparisons, with differences considered significant for *P* < 0.05. I–V relationships were constructed by determining current in response to NMDA/glycine at a range of holding potentials between − 100 and 0 mV in increments of 25 mV. Current was normalised to that at − 75 mV. Linear regression was carried out in GraphPad Prism 5 between − 50 and 0 mV and the X-intercept (extrapolated for GluN1-1A/2A) was considered the reversal potential.

## 3 Results

### 3.1 GluN3 altered NMDA receptor properties

**Fig. 2:**
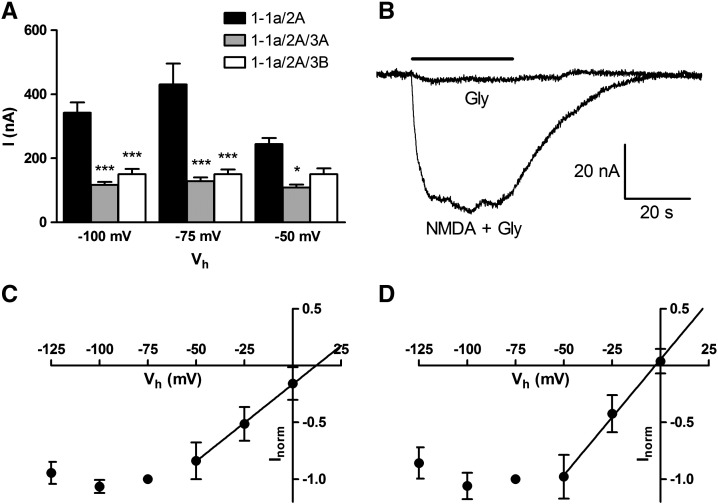
GluN3 subunits are incorporated into the NMDA receptors. A, Effect of subunit composition on steady-state current. Bars represent mean current (error bars are 95% CI, *n* = 40–85). Significant differences compared to GluN1-1a/2A within a voltage group are indicated above the bars (*P < 0.05 or ***P < 0.001, Bonferroni). B, Glycine does not activate a current in oocytes injected with GluN1-1a, GluN2A and GluN3A. Currents from the same oocyte exposed to glycine alone (1 mM) or NMDA/glycine (100/10 μM). Similar results were obtained from six oocytes from three different batches (V_*h*_ = − 50 mV). C,D, I/V relationships for oocytes injected with GluN1-1a/2A (C) or GluN1-1a/2A/3A (D). Current was evoked by application of 100 μM NMDA plus 10 μM glycine, normalised to that at − 75 mV and plotted as mean ± S.E.M. Points between − 50 mV and 0 mV are fit by linear regression.

The mean steady‐state current in response to 100 μM NMDA plus 10 μM glycine showed a significant reduction when GluN3A or GluN3B were co-injected with GluN1-1a and GluN2A (Fig. 2A). Oocytes injected with GluN1-1a, GluN2A and GluN3 subunits were tested with 1 mM glycine alone or with 1 mM Zn^2+^ but no significant current was recorded in three different batches of oocytes; both NMDA and glycine were required to elicit a response (Fig. 2B). When I/V relationships were produced the reversal potential for GluN1-1a/2A was 12.15 mV and this was reduced for GluN1-1a/2A/3A containing receptors to − 2.65 mV (Fig. 2C, D). Concentration–response relationships for NMDA (in the presence of 10 μM glycine) from oocytes injected with GluN1-1a/2A, GluN1-1a/2A/3A or GluN1-1a/2A/3B were virtually superimposed, however, equivalent plots for glycine (in the presence of 100 μM NMDA) show curves shifted to the left for NMDA receptors containing GluN3A or GluN3B (Supplementary Fig. 2).

### 3.2 The presence of GluN3A or GluN3B increased the IC_50_ for Mg^2+^

**Fig. 3:**
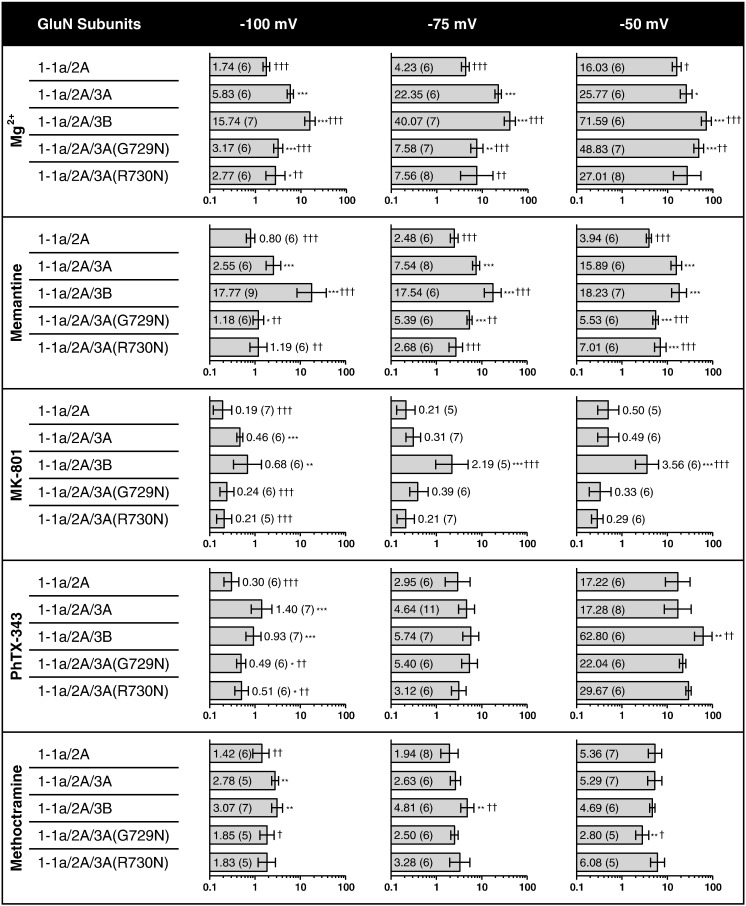
Summary of the IC_50_ values for Mg^2+^, memantine, MK-801, PhTX-343 and methoctramine block of the NMDA receptor subunit combinations tested. IC_50_s were obtained from the concentration–inhibition curves given in Figs. 4–8 for GluN1-1a/2A, GluN1-1a/2A/3A and GluN1-1a/2A/3B, and in Supplementary Fig. 3 for GluN1-1a/2A/3A(G729N) and GluN1-1a/2A/3A(R730N). Bars show IC_50_ (μM) ± 95% CI. Numbers in parentheses are the number of oocytes. Statistical comparisons were made with GluN1-1a/2A (*) or with GluN1-1a/2A/3A (^†^) with significance of difference accepted for *^,†^*P* < 0.05, **^,††^*P* < 0.01, ***^,†††^*P* < 0.001.

**Fig. 4:**
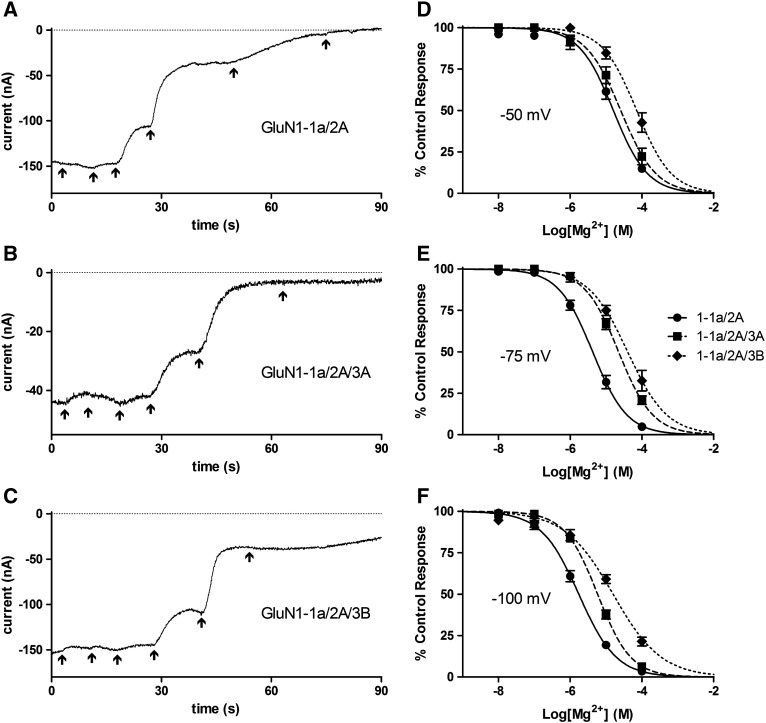
Characterising the block of GluN3-lacking and GluN3-containing NMDA receptors by Mg^2+^. A–C, sample traces of currents from *Xenopus* oocytes injected with GluN1-1A/2A (A), GluN1-1a/2A/3A (B) or GluN1-1a/2A/3B (C) and exposed to 100 μM NMDA plus 10 μM Gly with the addition of increasing concentrations of Mg^2+^ (10^− 8^ to 10^− 4^ M in 10-fold increments) indicated by the arrows, with the last arrow indicating wash. Traces begin 3 s before the lowest Mg^2+^ concentration was applied by which point a stable current was established; as this was after a variable period the first part of the trace is not shown. All traces were recorded at a V_h_ of − 75 mV. D–F, concentration–inhibition curves for Mg^2+^ block of 100 μM NMDA plus 10 μM Gly currents from *Xenopus* oocytes injected with GluN1-1A/2A (●, solid line), GluN1-1a/2A/3A (■, broken line) or GluN1-1a/2A/3B (♦, dotted line). Points are means ± S.E.M. for 6–7 oocytes. Curves are fits to the equation given in the Materials and methods section and IC_50_s derived from these are given in Fig. 3.

The IC_50_ values for Mg^2+^ inhibition of all subunit combinations tested are given in Fig. 3. Sample current traces at − 75 mV and concentration–inhibition curves are shown in Fig. 4. The presence of GluN3 subunits caused a significant increase in the IC_50_ for Mg^2+^ compared with GluN1-1a/2A alone, and for GluN1-1a/2A/3B it was found to be significantly higher than for GluN1-1a/2A/3A at all the voltages tested.

In order to explore the reasons why GluN3 subunits generally are capable of reducing the effectiveness of open‐channel block we prepared mutations at the N and N + 1 sites of GluN3A, sites known to be important for channel block, producing GluN3A(G729N) and GluN3A(R730N). At -100 mV both GluN3A mutations significantly reduced the IC_50_ compared with GluN3A wild-type (*P* < 0.0001 and *P* < 0.01 respectively), but it remained significantly higher than that for GluN1-1a/2A for both GluN1-1a/2A/3A(G729N) (*P* < 0.0001) and GluN1-1a/2A/3A(R730N) (*P* < 0.05). At − 75 mV and − 50 mV the GluN3A mutations resulted in an IC_50_ for Mg^2+^ where GluN1-1a/2A/3A(G729N) was again intermediate, being significantly lower than that for GluN1-1a/2A/3A (*P* < 0.0001) and significantly higher than that for GluN1-1a/2A (*P* < 0.01); while GluN1-1a/2A/3A(R730N) restored the IC_50_ to levels that were not significantly different to that for GluN1-1a/2A.

### 3.3 Memantine had a similar pattern of block to Mg^2+^

**Fig. 5:**
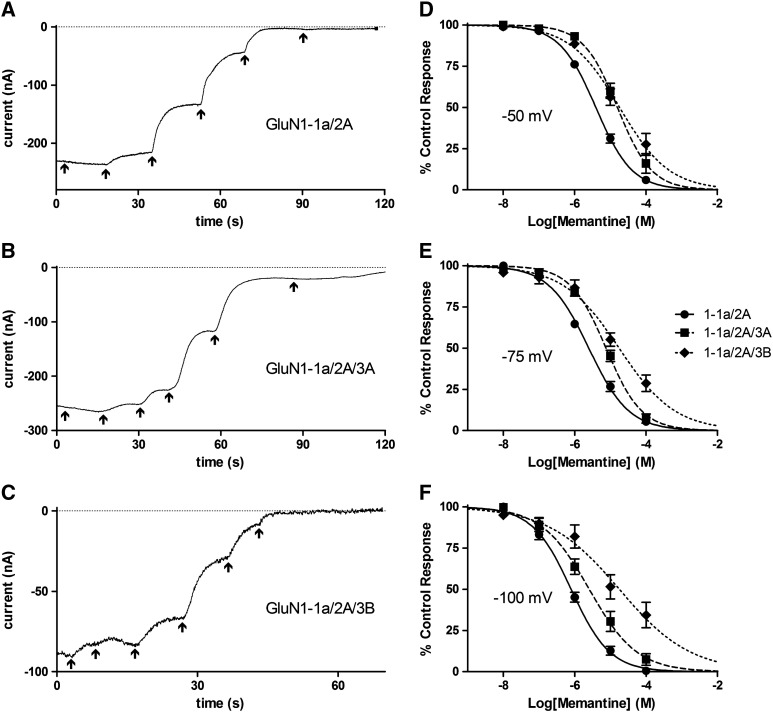
Characterising the block of GluN3-lacking and GluN3-containing NMDA receptors by memantine. A–C, sample traces of currents from *Xenopus* oocytes injected with GluN1-1A/2A (A), GluN1-1a/2A/3A (B) or GluN1-1a/2A/3B (C) and exposed to 100 μM NMDA plus 10 μM Gly with the addition of increasing concentrations of memantine (10^− 8^ to 10^− 4^ M in 10-fold increments) indicated by the arrows, with the last arrow indicating wash. Traces begin 3 s before the lowest memantine concentration was applied by which point a stable current was established; as this was after a variable period the first part of the trace is not shown. All traces were recorded at a V_h_ of − 75 mV. D–F, concentration–inhibition curves for memantine block of 100 μM NMDA plus 10 μM Gly currents from *Xenopus* oocytes injected with GluN1-1A/2A (●, solid line), GluN1-1a/2A/3A (■, broken line) or GluN1-1a/2A/3B (♦, dotted line). Points are means ± S.E.M. for 6–9 oocytes. Curves are fits to the equation given in the Materials and methods section and IC_50_s derived from these are given in Fig. 3.

The IC_50_ values for memantine inhibition of all subunit combinations tested are given in Fig. 3. Sample current traces at − 75 mV and concentration–inhibition curves are shown in Fig. 5. The IC_50_ for memantine at − 100 and − 75 mV was significantly increased for GluN3-containing NMDA receptors compared with GluN1-1a/2A, with that for GluN1-1a/2A/3B again being significantly higher than that for GluN1-1a/2A/3A (*P* < 0.0001). A similar increase was found at − 50 mV, but at this voltage the IC_50_s for GluN1-1a/2A/3A and GluN1-1a/2A/3B were not significantly different to each other.

At − 100 and − 75 mV GluN1-1a/2A/3A(G729N) led to a reduction in IC_50_ which became significantly lower than that for GluN1-1a/2A/3A (*P* < 0.01) while remaining significantly higher than that for GluN1-1a/2A (*P* < 0.05 at − 100 mV; *P* < 0.0001 at − 75 mV); GluN3A(R730N) completely restored block so that the IC_50_ for memantine was not significantly different to that for GluN1-1a/2A. At − 50 mV both mutations led to an intermediate IC_50_ that was both significantly lower than that for GluN1-1a/2A/3A (*P* < 0.0001) and significantly higher than that for GluN1-1a/2A (*P* < 0.01).

### 3.4 MK-801 IC_50_ was mostly influenced by GluN3B

**Fig. 6:**
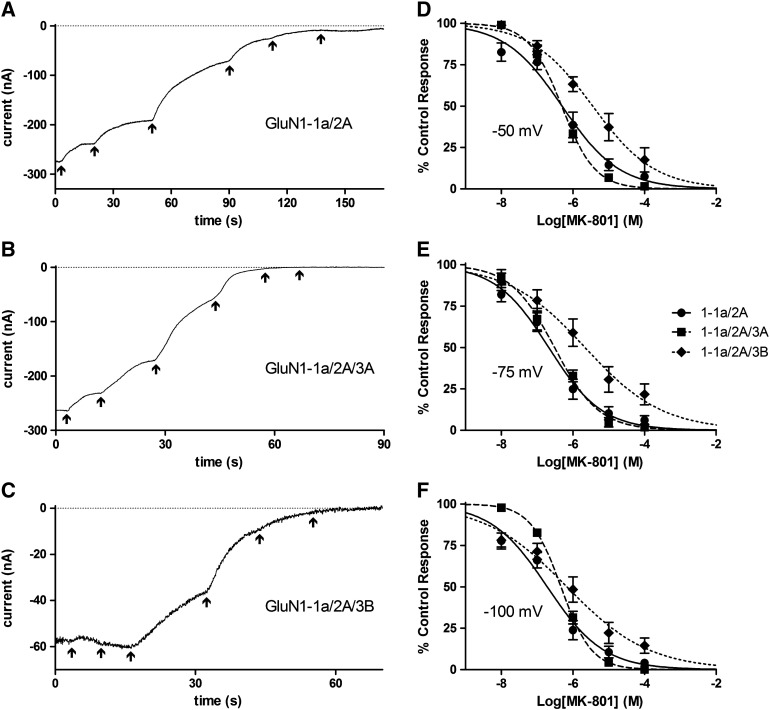
Characterising the block of GluN3-lacking and GluN3-containing NMDA receptors by MK-801. A–C, sample traces of currents from *Xenopus* oocytes injected with GluN1-1A/2A (A), GluN1-1a/2A/3A (B) or GluN1-1a/2A/3B (C) and exposed to 100 μM NMDA plus 10 μM Gly with the addition of increasing concentrations of MK-801 (10^− 8^ to 10^− 4^ M in 10-fold increments) indicated by the arrows, with the last arrow indicating wash. Traces begin 3 s before the lowest MK-801 concentration was applied by which point a stable current was established; as this was after a variable period the first part of the trace is not shown. All traces were recorded at a V_h_ of − 75 mV. D–F, concentration–inhibition curves for MK-801 block of 100 μM NMDA plus 10 μM Gly currents from *Xenopus* oocytes injected with GluN1-1A/2A (●, solid line), GluN1-1a/2A/3A (■, broken line) or GluN1-1a/2A/3B (♦, dotted line). Points are means ± S.E.M. for 5–7 oocytes. Curves are fits to the equation given in the Materials and methods section and IC_50_s derived from these are given in Fig. 3.

IC_50_ values for MK-801 inhibition of all subunit combinations tested are given in Fig. 3. Sample current traces at − 75 mV and concentration–inhibition curves are shown in Fig. 6. At − 100 mV MK-801 had an IC_50_ at GluN1-1a/2A which was significantly lower than that for GluN1-1a/2A/3A (*P* < 0.01) and GluN1-1a/2A/3B (*P* < 0.01), but the IC_50_s for the latter two combinations were not significantly different to each other. At − 75 and − 50 mV the IC_50_ for MK-801 at GluN1-1a/2A/3B was significantly higher than GluN1-1a/2A and GluN1-1a/2A/3A (*P* < 0.0001) but there was no significant difference between the IC_50_s of the latter two combinations.

At − 100 mV both the GluN3A mutations completely restored block by MK-801 as the IC_50_ values were not significantly different to GluN1-1a/2A. Since GluN1-1a/2A/3A did not increase the IC_50_ at − 75 mV and − 50 mV the mutations led to no change in potency except for GluN3A(R730N) at − 50 mV, where it actually increased the potency and led to an IC_50_ that was significantly lower than that for GluN1-1a/2A (*P* < 0.01).

### 3.5 PhTX-343 block was only sensitive to GluN3 subunits at more negative V_h_

**Fig. 7:**
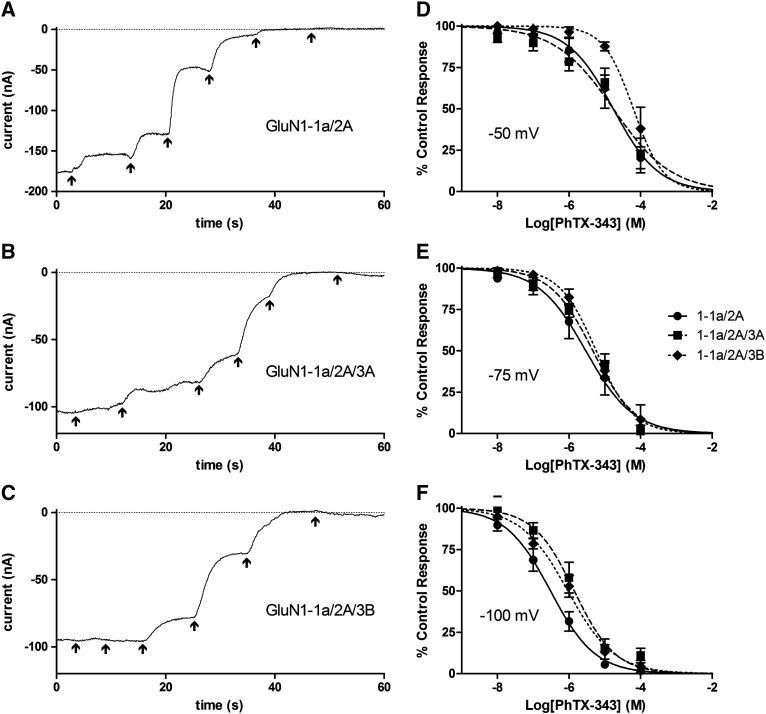
Characterising the block of GluN3-lacking and GluN3-containing NMDA receptors by PhTX-343. A–C, sample traces of currents from *Xenopus* oocytes injected with GluN1-1A/2A (A), GluN1-1a/2A/3A (B) or GluN1-1a/2A/3B (C) and exposed to 100 μM NMDA plus 10 μM Gly with the addition of increasing concentrations of PhTX-343 (10^− 8^ to 10^− 4^ M in 10-fold increments) indicated by the arrows, with the last arrow indicating wash. Traces begin 3 s before the lowest PhTX-343 concentration was applied by which point a stable current was established; as this was after a variable period the first part of the trace is not shown. All traces were recorded at a V_h_ of − 75 mV. D–F, concentration–inhibition curves for PhTX-343 block of 100 μM NMDA plus 10 μM Gly currents from *Xenopus* oocytes injected with GluN1-1A/2A (●, solid line), GluN1-1a/2A/3A (■, broken line) or GluN1-1a/2A/3B (♦, dotted line). Points are means ± S.E.M. for 6–11 oocytes. Curves are fits to the equation given in the Materials and methods section and IC_50_s derived from these are given in Fig. 3.

IC_50_ values for PhTX-343 inhibition of all subunit combinations tested are given in Fig. 3. Sample current traces at − 75 mV and concentration–inhibition curves are shown in Fig. 7. At -100 mV the IC_50_ for PhTX-343 was significantly increased for GluN1-1a/2A/3A and GluN1-1a/2A/3B compared with GluN1-1a/2A (*P* < 0.0001), while the IC_50_s for the GluN3A- or GluN3B-containing receptors were not significantly different to each other. At − 75 and − 50 mV there were no significant differences found in the IC_50_ for PhTX-343 for all the subunits tested, except for that of GluN1-1a/2A/3B which was significantly higher than that of GluN1-1a/2A and GluN1-1a/2A/3A at − 50 mV (*P* < 0.01).

At − 100 mV the GluN3A mutations partially restored the IC_50_ for PhTX-343 which was significantly reduced compared with GluN1-1a/2A/3A (*P* < 0.01) but remained significantly higher than GluN1-1a/2A (*P* < 0.05).

### 3.6 Methoctramine inhibition was minimally affected by GluN3 subunits

**Fig. 8:**
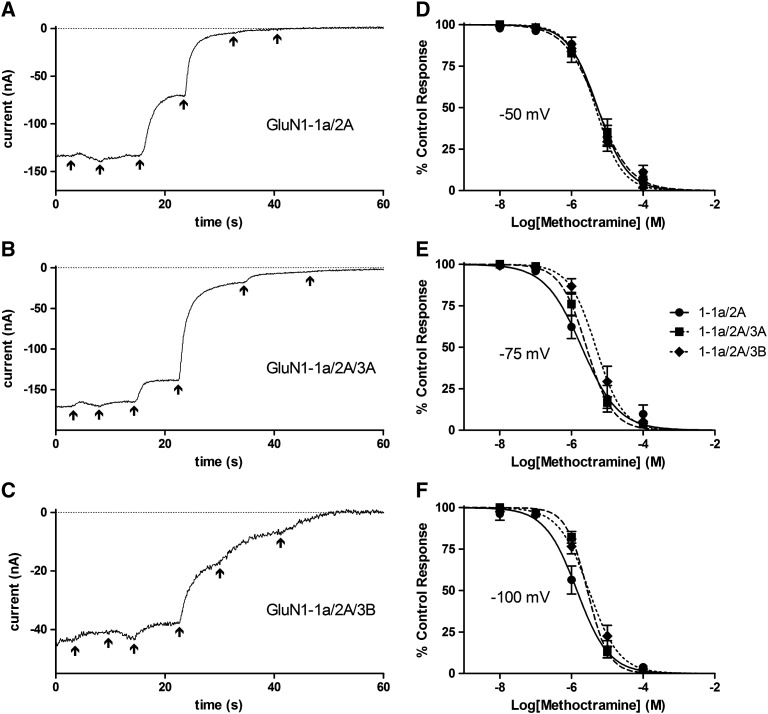
Characterising the block of GluN3-lacking and GluN3-containing NMDA receptors by methoctramine. A–C, sample traces of currents from *Xenopus* oocytes injected with GluN1-1A/2A (A), GluN1-1a/2A/3A (B) or GluN1-1a/2A/3B (C) and exposed to 100 μM NMDA plus 10 μM Gly with the addition of increasing concentrations of methoctramine (10^− 8^ to 10^− 4^ M in 10-fold increments) indicated by the arrows, with the last arrow indicating wash. Traces begin 3 s before the lowest methoctramine concentration was applied by which point a stable current was established; as this was after a variable period the first part of the trace is not shown. All traces were recorded at a V_h_ of − 75 mV. D-F, concentration–inhibition curves for methoctramine block of 100 μM NMDA plus 10 μM Gly currents from *Xenopus* oocytes injected with GluN1-1A/2A (●, solid line), GluN1-1a/2A/3A (■, broken line) or GluN1-1a/2A/3B (♦, dotted line). Points are means ± S.E.M. for 5–8 oocytes. Curves are fits to the equation given in the Materials and methods section and IC_50_s derived from these are given in Fig. 3.

IC_50_ values for methoctramine inhibition of all subunit combinations tested are given in Fig. 3. Sample current traces at − 75 mV and all concentration–inhibition curves are shown in Fig. 8. At − 100 mV there was a small but significant increase in the methoctramine IC_50_ for GluN1-1a/2A/3A (*P* < 0.01) and GluN1-1a/2A/3B (*P* < 0.01) when compared with GluN1-1a/2A but it was not different when compared to each other. At − 75 mV the IC_50_ for GluN1-1a/2A/3B remained significantly higher than that for both GluN1-1a/2A and GluN1-1a/2A/3A (*P* < 0.01). At − 50 mV there were no significant differences in the IC_50_s for methoctramine between GluN1-1a/2A, GluN1-1a/2A/3A and GluN1-1a/2A/3B.

At − 100 mV the mutations led to the IC_50_ for methoctramine being completely restored so that they were now not significantly different to that for GluN1-1a/2A. At − 75 mV, since the IC_50_ for methoctramine at GluN1-1a/2A/3A was now not significantly different to that for GluN1-1a/2A the GluN3A mutations had no effect. At − 50 mV, the GluN3A(G729N) mutation led to an IC_50_ value that was significantly lower than that for GluN1-1a/2A (*P* < 0.01).

## 4 Discussion

GluN3 subunits have been frequently reported to have a negative impact on NMDA mediated current and serves as an indicator of their expression and incorporation into a functional NMDA receptor. In this study, both GluN3A and GluN3B subunits reduced the mean amplitude of NMDA/glycine evoked currents, presumably because of the previously reported reduced single channel current that may be due to the charged R residue altering Ca^2+^ permeability, which is further supported by the reduction in the reversal potential (Cavara et al., 2010; Sasaki et al., 2002). Additionally, the sensitivity to glycine was increased when the GluN3A or GluN3B subunits were co-expressed with GluN1-1a and GluN2A. This change is consistent with previous reports of the higher affinity of the GluN3A ligand binding domain to glycine and again confirmed their presence in the expressed NMDA receptor (Yao and Mayer, 2006). There have been reports of a GluN1/3 receptor population when GluN1, 2 and 3 were injected into *Xenopus* oocytes, however such studies are limited by the fact that oocytes express endogenous *Xen*GluN2B (Ulbrich and Isacoff, 2008). In our study glycine alone or together with Zn^2+^ failed to elicit a significant response. Glycine activated currents are thought to be rapidly desensitising, although responses have been shown to be visible over a period of 10 s meaning our experimental set-up would have detected them (Madry et al., 2008). The lack of response may be because we were using GluN1-1a which is a poor former of functional GluN1/3 receptors (Smothers and Woodward, 2009). The fact that our concentration–inhibition curves for the open-channel blockers were not obviously biphasic, while showing reproducible changes in IC_50_, led us to conclude that the co-expression of GluN1, 2 and 3 led to a major population of triheteromeric GluN1-1a/2A/3 receptors. However, we cannot rule out the presence of a population of diheteromeric GluN1-1a/2A receptors, in which case the effects of GluN3 subunits on receptor properties reported here would be underestimated.

Mg^2+^, memantine and MK-801 are well established open-channel blockers of NMDA receptors and studies of their binding sites show an overlap at the N and N + 1 sites, but also at some other residues in the channel pore and vestibule (Kashiwagi et al., 2002). Our experiments showing that GluN3A and GluN3B generally reduced block by Mg^2+^, memantine and MK-801 led us to the hypothesis that the differences in GluN3 subunits at the N site and N + 1 site may be responsible for this. However, the mutations GluN3A(G729N) and GluN3A(R730N) did not always completely restore the blocking potency, therefore, residues other than those at the N and N + 1 sites may play a part. In support of our findings it has been shown that in glycine-gated GluN1-3a/3B receptors, mutation of the GluN3B N site or N + 1 site to N only partially restored block by 0.5 mM Mg^2+^ (Cavara et al., 2010). However, we cannot completely rule out the possibility that the mutations reduce the ability of the GluN3A subunit to incorporate into a channel complex, although in this situation we would expect a consistent impact across all of the tested compounds.

Further residues have been identified on GluN1 and GluN2 subunits that play a role in open-channel block. The W residue at position − 8 (Fig. 1A) in GluN1 and GluN2 influences Mg^2+^ block, but mutations here only resulted in a loss of activity if there was a loss of the aromatic ring; this would be lost with GluN3A and GluN3B as they have N at this position (Williams et al., 1998). Also, W-8 of GluN2B, when mutated to N, which is found at that position in GluN3A and GluN3B, caused a large drop in potency for several channel blockers including memantine and MK-801 (Kashiwagi et al., 2002) and is therefore another likely candidate for reduced block of GluN3-containing channels.

The binding sites for PhTX-343 and methoctramine on NMDA receptors have not yet been explored in detail. The strong voltage-dependence of PhTX-343 inhibition shown here and in previous studies (Mellor et al., 2003) implies that it has a binding site deep in the pore while the weaker voltage-dependence of methoctramine inhibition implies a shallower site in the pore. Studies of PhTX-343 with nicotinic acetylcholine receptors and AMPA receptors suggest that the polyamine chain interacts with deep pore residues such as the hydrophilic and intermediate negatively charged rings of nicotinic acetylcholine receptors or residues beyond the Q/R site of AMPA receptors (Andersen et al., 2006; Brier et al., 2003; Tikhonov et al., 2002, 2004). The more hydrophobic headgroup of PhTX-343 is then thought to interact with residues in the wider outer parts of the pore, i.e. outer M2 and M1 residues in nicotinic acetylcholine receptors and M3 residues in AMPA receptors. It is likely that PhTX-343 inhibits NMDA receptors with a similar binding mode. In AMPA receptors, the Q/R site has been shown to be important for the activity of polyamine toxins such as PhTX-343 and for block by internal spermine that causes inward rectification, as an R at the Q/R site on the GluA2 subunit (Fig. 1A) of the AMPA receptor results in receptors that are not blocked by polyamines (Brackley et al., 1993; Mellor, 2010; Strømgaard and Mellor, 2004). However, we found that this was not entirely the case with the R at the N + 1 site of GluN3 as the loss of activity by PhTX-343 was only 4.67-fold at most. It may be that the R + 1 side-chain is less exposed to the pore and therefore has less impact; it is known that in pore-loop channels the lining may be generated by main chain carbonyl oxygens rather than the amino acid side-chains (Tikhonov et al., 2002). At NMDA receptors the polyamine tail of anthraquinone and anthracene polyamines has been shown to interact with the negatively charged E at position + 5 of GluN1 (Fig. 1A), located beyond the N site (Jin et al., 2007). The GluN3A and GluN3B subunits have I or S in this position which could be interfering with this binding. This difference may only manifest itself at highly negative potential due to its deep position within the pore; as we demonstrated here the distinction was lost as the V_h_ became less negative. Block by anthraquinone compounds was also sensitive to mutations at N, N + 1, W-8 and numerous other sites in the outer pore (Jin et al., 2007).

Methoctramine inhibition had limited voltage-dependence and was minimally affected by the introduction of GluN3 subunits or mutation of G729 and R730, i.e. unlike PhTX-343 it remained potent across the voltage and subunit range tested. Presumably its binding site is not as deep as the other compounds meaning it would not be influenced to the same degree by changes at the N and N + 1 site, and this was most obvious at highly negative V_*h*_.

Perhaps the most significant finding from this study was that GluN3B generally provided more resistance than GluN3A to block by Mg^2+^, memantine and MK-801, and perhaps PhTX-343 at less negative V_h_. This cannot be explained by the substitutions at the N and N + 1 sites investigated in this study because both GluN3A and GluN3B have G and R at the N and N + 1 sites. There are several differences between the GluN3A and GluN3B sequences in the M2-M3 region that define the narrow part and vestibule of the pore. For example, W/P (GluN3A/B) at + 11, C/V at + 27, E/D at + 42 and I/T at + 44 within the M3 alpha helix (Fig. 1A) may affect binding directly, or indirectly through altered gating properties, leading to differences between GluN3A and GluN3B (Cordes et al., 2002). Other regions such as the agonist binding domain, the N-terminal domain and all the linker regions may be involved as these have been shown to impact on Mg^2+^ and memantine potency in GluN1/GluN2 containing NMDA receptors (Wrighton et al., 2008; Yuan et al., 2009). An approach using chimeric GluN3A/B subunits, similar to that used by Wrighton et al. (2008), may be useful in identifying these influential regions.

In summary, the current study has confirmed that the GluN3 subunits can reduce the potency of well categorised open-channel blockers and has further shown that GluN3B is generally more effective than GluN3A. The changes can be largely explained by the presence of G and R at the N and N + 1 sites for GluN3A but other residues in GluN3B need to be explored to explain its higher resistance to channel block.
