# Supplementary material for: Open-channel blockade is less effective on GluN3B than GluN3A subunit-containing NMDA receptors
Source: Eur J Pharmacol. 2012 Jul 5;686(1-3):22–31. doi: 10.1016/j.ejphar.2012.04.036 (PMC3657159; doi:10.1016/j.ejphar.2012.04.036)
Supplement: Supplementary file 1 — Supplementary materials [file mmc1.docx]

**Supplementary Figure 1**. Application of an inactive compound using the sequential application regime described in the Methods section, to an oocyte expressing GluN1-1a/2A, voltage clamped at -75 mV and exposed to 100 µM NMDA plus 10 µM glycine. The current remains stable during switching of the solutions over an extended period of time.

**

**

**Supplementary Figure 2:** Concentration-response relationships for NMDA (A) or glycine (B) activated currents in Xenopus oocytes injected with GluN1-1a/2A (⚫), GluN1-1a/2A/3A (◼) or GluN1-1a/2A/3B (▲). Points are mean ± SEM from 3-7 oocytes. The dip in response at the highest concentration of NMDA or glycine is likely to be due to desensitization. Approximations of EC_50_ values for NMDA are 14 µM for all receptors, and for glycine are 1.7, 0.34 and 0.43 µM for GluN1-1a/2A, GluN1-1a/2A/3A and GluN1-1a/2A/3B respectively.

**

**

**Supplementary Figure 3:** Concentration-inhibition curves for inhibition of 100 µM NMDA/10 µM glycine evoked currents from oocytes injected with GluN1-1a/2A (⚫), GluN1-1a/2A/3A (◼), GluN1-1a/2A/3A(G729N) (▲) or GluN1-1a/2A/3A(R730N) (▼) by Mg^2+^ (**A**), memantine (**B**), MK-801 (**C**), PhTX-343 (**D**) or methoctramine (**E**) at holding potentials of -50 mV (left), -75 mV (centre) or -100 mV (right). The data are plotted as mean % of control response ± S.E.M. (n = 5-11) and fit with a Hill equation. IC_50_s are given in Table 1.
